# The future of tumor vaccines in the post‐COVID‐19 era—Current challenges

**DOI:** 10.1002/iid3.521

**Published:** 2021-09-02

**Authors:** Panagiotis F. Christopoulos

**Affiliations:** ^1^ Department of Pathology, Section of Research Oslo University Hospital Rikshospitalet and University of Oslo Oslo Norway

**Keywords:** cancer vaccines, COVID‐19, immunotherapy, SARS‐CoV‐2

The recent coronavirus (2019) outbreak has emerged as the biggest crisis of our time, reflecting outrageous human losses and unprecedented social, psychological, and economic consequences, globally. Since the release of the SARS‐CoV‐2 genome,^1^ the race for vaccine development has resulted in various licensed regimens using different approaches and technologies. With cancer still prevailing as the deadliest—compared to COVID‐19—disease (http://gco.iarc.fr/today/fact-sheets-cancers), it is most likely that the recent advances in vaccine design will find their way in tumor immunotherapeutics, in the near future. Given the similarities and differences in the immune responses against viral infections or cancer, it is anticipated that some of the current approaches might hold stronger potentials than others.

Although the end goal is the same—to mount a specific immune response against the spike protein of SARS‐CoV‐2—the current vaccine technologies (as of July 2021) use three different approaches to “deliver” the viral antigen: (i) messenger RNA (mRNA), (ii) viral vectors, or (iii) protein subunits.^2^, ^3^, ^4^, ^5^, ^6^ To date, even though all of these approaches have shown impressive efficacy against the coronavirus, each presents with pros and cons which might be differently attributed to the cancer setting.

In this vein, the *type of vaccine‐induced immunity* is a key determining factor. In contrast to antiviral immunity, where antibodies can efficiently reduce the viral titers (via marking for destruction and/or inhibiting infectivity), humoral immune responses may have a secondary/supplementary role in antitumor immunity, since are restricted to cell‐surface antigens only. Though all current approaches against SARS‐CoV‐2 could potentially induce both humoral and cellular immunity, data on CD8^+^ responses are still scarce and inconclusive. Previous evidence points to strong cellular responses induced by viral vectors, against infectious diseases and cancer,^7^ in contrast, to favorably antibody responses by protein‐in adjuvants vaccines against extracellular pathogens.^8^ It is anticipated that vaccine‐induced long‐lasting cytotoxic T lymphocyte responses, will be detrimental in the war against cancer.

*Selective yet broad immunogenicity* of various antigens is another fundamental area. Unlike the foreign antigens of SARS‐CoV‐2, mutation events of self‐proteins during carcinogenesis give rise to the tumor's neoantigens. Not only the mutational burden differs greatly between the different tumor types but also individual tumors might be highly heterogeneic. In this regard, nucleic acid vaccine technology (mRNA in lipid nanoparticles) allows for the encoding of multiple and full‐length antigens leading to the presentation and cross‐presentation of multiple epitopes and eventually epitope spreading and thus will likely have the advantage against different cancer types and heterogeneous tumors. Generation of a broad tumor‐specific T‐cell repertoire is of high priority.

*Adaptation and personalization*. New, individual passenger mutations continuously accumulate during cancer progression and metastasis. As such, rapid and highly adaptable vaccine platforms, able to meet the ongoing challenges of chronic disease, will be in the foreground. Despite the unfortunate conditions created by the current pandemic, science is at its most exciting times; lessons to be learned from the future adaptation of the current vaccine platforms against the different rising variants (e.g., the new B.1.617 lineages^9^) will be used against tumors accumulating novel mutations and/or developing tumors that eliminated the clones bearing the targeted antigen under immune pressure (immunoediting). Due to their design, the mRNA vaccine formulations are probably better suited for these modifications (newly and rapidly synthesized mRNAs in particle carriers).

*Innate immunity and inflammation* are also not of lesser importance. While protein vaccines are usually conjugated with adjuvants, viral vectors and mRNA vaccines can per se activate pattern recognition receptors, leading to type I IFN production and secretion of proinflammatory cytokines. Even though innate immune cell activation via danger/pathogen recognition is required for adaptive immunity (costimulation), it is also a double edged sword in some cases, since multiple toll‐like receptors (TLRs) activation and RNA sensing might lead to mRNA degradation and block of translation and antigen presentation,^10^ as well as inhibition of CD8^+^ immunity,^11^ collectively known as the type I IFN paradox. On the other hand, aberrant activation of innate immune cells might lead to hyperinflammation and severe—even life‐threatening in some cases—systemic complications. Timing and kinetics seem to regulate these controversial events. It is, however, likely that a controlled (nonsystemic) inflammation able to support the cancer‐immunity cycle and counterbalance the immunosuppressive signals of the tumor microenvironment—turning cold tumors to hot—will prove a powerful weapon in the arsenal.

*Production costs and other challenges*. Traditionally, cellular tumor therapies are based on autologous dendritic cells (DCs) isolation, expansion, education (using one's neoantigens), maturation (cytokines and TLR ligands cocktails), and reinfusion, which practically reflects considerable time and cost limitations since there is a need to reinvent the vaccine for each patient. Even though personalization might still hold true for the neoantigens part, a universal platform for antigen delivery possess significant advances over the autologous DCs. Depending on the approach, several other challenges and considerations remain, like the vast production and purification costs of protein antigens required for the protein subunits formulations or the potential risk of additional mutations of DNA‐based technologies (insertional mutagenesis). Again, mRNA vaccines are in the spotlight.

*Combination with other regimens*. Even if a successful antitumor response is initiated, cancer cells recruit different mechanisms to evade T‐cell recognition and destruction, including T‐cell exhaustion. In this vein, a combination of the tumor's neoantigens‐based vaccine with immune checkpoint blockade (ICB) therapy (anti‐PD‐1/PD‐L1), is highly anticipated.

Life in the post‐COVID‐19 era might never be the same again. But every cloud has a silver lining; the current crisis has highlighted among others, forgotten values, like the importance of health sciences and medical research, as well as the need for large‐scale health care infrastructures. As we move closer to the end of this pandemic, the gain of knowledge and technologies developed will naturally be used against future challenges or other long‐standing threats, like cancer. Even though different scenarios might apply to the cancer setting, experience during this crisis proved the feasibility and rapid massive production of therapeutic vaccines; from basic research to clinical application, in record time. Multi‐antigen delivery leading to broad T‐cell repertoire and innate immune activation supporting the Th1 responses are the key parameters governing the translation efficacy of newly developed tumor vaccines. Current immunotherapies—ICB and chimeric antigen receptor T cells—have revolutionized cancer treatment, though there is still plenty of room for improvements. With lessons learned from COVID‐19, is the dawn of a cancer‐free era just around the corner?

## CONFLICT OF INTEREST

The author declares no conflict of interest.
